# Strain tracking in complex microbiomes using synteny analysis reveals per-species modes of evolution

**DOI:** 10.1038/s41587-024-02276-2

**Published:** 2024-06-19

**Authors:** Hagay Enav, Inbal Paz, Ruth E. Ley

**Affiliations:** 1 Department of Microbiome Science, Max Planck Institute for Biology, Tübingen, Germany; 2 Cluster of Excellence EXC 2124: Controlling Microbes to Fight Infections (CMFI), University of Tübingen, Tübingen, Germany

**Keywords:** Bacterial genomics, Bioinformatics, Genome informatics

## Abstract

Microbial species diversify into strains through single-nucleotide mutations and structural changes, such as recombination, insertions and deletions. Most strain-comparison methods quantify differences in single-nucleotide polymorphisms (SNPs) and are insensitive to structural changes. However, recombination is an important driver of phenotypic diversification in many species, including human pathogens. We introduce SynTracker, a tool that compares microbial strains using genome synteny—the order of sequence blocks in homologous genomic regions—in pairs of metagenomic assemblies or genomes. Genome synteny is a rich source of genomic information untapped by current strain-comparison tools. SynTracker has low sensitivity to SNPs, has no database requirement and is robust to sequencing errors. It outperforms existing tools when tracking strains in metagenomic data and is particularly suited for phages, plasmids and other low-data contexts. Applied to single-species datasets and human gut metagenomes, SynTracker, combined with an SNP-based tool, detects strains enriched in either point mutations or structural changes, providing insights into microbial evolution in situ.

## Abstract (web summary)

The evolution of microbial strains is revealed by analyzing structural variation.

## Main

Microbiomes are assemblages of diverse microbial species that often contain a vast diversity of strains. Within any given species, strain variation can arise from in situ evolution through several mechanisms, including the accumulation of point mutations and structural changes to the genome arising from insertions, deletions, horizontal gene transfers and intragenomic rearrangements requiring recombination. Species can differ in the main mode of genomic changes underlying strain diversification in their habitat. For instance, in the human gut, strains of *Bacteroides* *fragilis* evolve mainly through the accumulation of point mutations but not through recombination^1^. In contrast, *Helicobacter* *pylori* owes its strain diversity to both recombination and the accumulation of point mutations^2^. Point mutations and structural changes can operate on different parts of the same genome, as has been shown for many fungal phytopathogens^3^. Mutations and structural variations alike can lead to important functional differences between strains. For *H*. *pylori*, a single point mutation is associated with the development of gastric cancer^4^; for *Staphylococcus* *aureus*, a single point mutation is associated with jumping hosts from humans to rabbits^5^. Recombination has been shown to drive antibiotic resistance in *Streptococcus* *pneumoniae*^6^ and virulence in *Neisseria* *meningitidis*^7^. Other bacterial pathogens use recombination to generate phase variation important for immune evasion^8–10^.

Despite the importance of structural changes to the genome in driving strain diversity, the majority of tools used to date to compare strains (as reviewed in the literature^11^) use a single approach based on pairwise comparisons of single-nucleotide polymorphisms (SNPs) between genomes.

SNP-based genome comparison tools are also used to assign genomes to the same or different strain of a species using similarity thresholds. SNP-based tools are highly sensitive to mutation-driven diversity but are relatively insensitive to structural differences between genomes, particularly if they are based on the mapping of reads to reference genomes^11^. This bias could lead to underestimation of strain diversity in highly recombining species. SNP-based methods may also potentially overestimate genomic diversity because of technical or biological factors, such as sequencing errors or the emergence of hypermutators with elevated point-mutation rates^12,13^. These aspects alone motivate the development of tools that capture structural differences between genomes. Moreover, when used in a comparative manner with SNP-based approaches, such tools promise to reveal a more complete view of how different species generate strain diversity.

Gene synteny has been used previously to estimate evolutionary distances between genomes of eukaryotes^14–16^; in microorganisms, gene synteny was used to identify horizontal gene transfer events^17^. However, microsynteny (the local conservation of genetic-marker order in genomic regions) is an overlooked component of variation for assessing genomic diversity^18^ and constitutes an untapped and rich source of genomic information for microbial strain comparisons. Related strains should share syntenic regions, regardless of SNP variation between homologous genes.

Here, we developed SynTracker, a tool that uses microsynteny to compare strains within species. SynTracker uses pairwise comparisons of homologous genomic regions in either metagenomic or genomic assemblies, followed by scoring the average synteny per pair of strains (average pairwise synteny score, APSS). SynTracker does not require a precompiled reference genome database but instead uses only a single reference genome per species of interest and it is relatively insensitive to SNPs. We designed SynTracker to give lower weight to SNPs and higher weight to insertions, deletions and recombination events. These genomic differences are less abundant than SNPs and are less likely to result from sequencing errors^19^.

We applied SynTracker to bacterial isolate genomes and to human gut metagenome datasets from mother–infant pairs (MIPs) sampled in different geographic locations and benchmarked it against existing strain-tracking tools. SynTracker outperformed existing tools in terms of both accuracy and sensitivity (that is, the total number of strain pairs identified), making it particularly suited for the analysis of low-abundance species, plasmids and phages. When used in combination with an SNP-based tool, SynTracker allowed the identification of hypermutators (large differences in SNPs but low levels of recombination, insertions and deletions) and hyper-recombinators (low differences in SNPs but high levels of genome structural variations) both within single-species datasets and a human gut metagenome. SynTracker is available as an open-source program on GitHub (https://github.com/leylabmpi/SynTracker).

## Results

### SynTracker compares strains within species using metagenome or genome data

SynTracker identifies synteny blocks in pairs of homologous genomic regions derived from isolate genomes, metagenomic assemblies or metagenome-assembled genomes (MAGs). As input, the pipeline accepts one genome per species of interest (bacterium, phage or plasmid), either fully or partially assembled, to be used as a reference and a collection of metagenomic assemblies (or genomes, if genomes are to be compared).

#### Step 1: identification of homologous regions

**Fig. 1:**
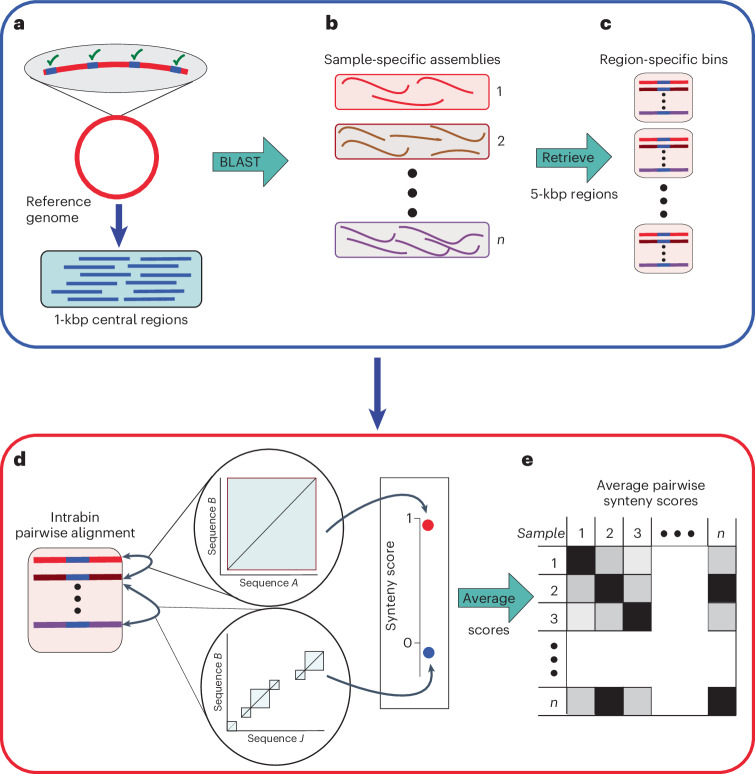
Illustration of the SynTracker algorithm. **a**, The reference genome is fragmented to yield central regions, that is, 1-kbp-long regions located 4 kbp apart. **b**, Each central region is used as a query for a BLAST search against a collection of sample-specific assemblies (or genomes, as appropriate). **c**, BLAST hits are retrieved with 2 kbp on each side of the hit; however, this can be modified by the user. All bins resulting from the same BLAST search are placed in the same region-specific bin. **d**, Within each bin, an all-versus-all pairwise alignment is performed to identify synteny blocks in pairs of sequences. Synteny scores are calculated on the basis of the number of blocks and the sum of the length of the blocks. **e**, For each pair of samples (or genomes) *n* regions are sampled and their synteny scores are averaged to yield the APSS.

The reference genome is fragmented to create a collection of 1-kbp genomic regions, located 4 kbp apart (‘central regions’; Fig. 1a). Next, we convert the collection of per-sample metagenomic assemblies (or genomes) to a basic local alignment search tool (BLAST)^20^ database and use the central regions as queries for a high-stringency nucleotide BLAST (BLASTn) search (identity = 97%, minimal query coverage = 70%; Fig. 1b) to minimize the possibility of receiving multispecies hits or hits located within regions with high copy-number variation. For each BLAST hit, we then retrieve the target sequence and the flanking 2-kbp regions upstream and downstream of the target sequence. This strategy results in high specificity when identifying homologs to the central regions, while allowing for high variance in the sequence composition of the flanking regions. These parameters can be modified by the user, according to preferences.

#### Step 2: calculation of region-specific synteny scores

**Extended Data Fig. 1:**
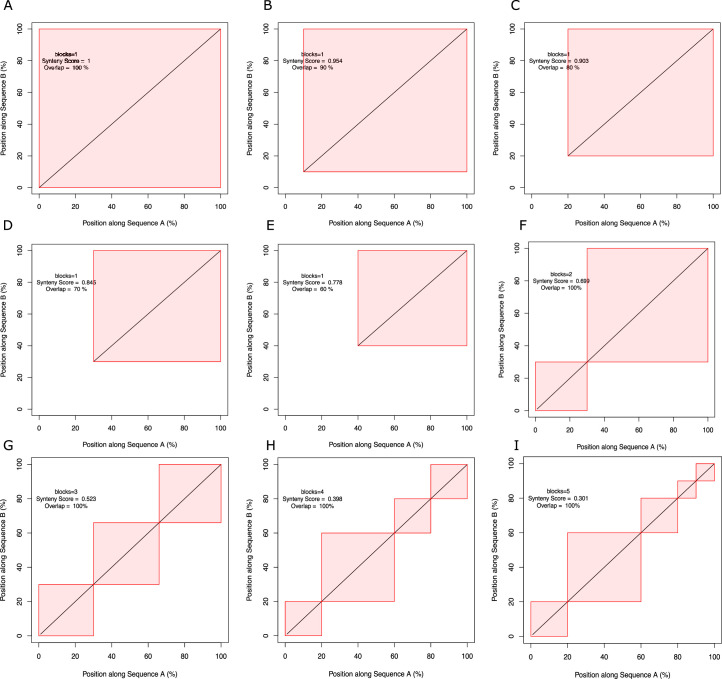
Examples for the influence of the number of synteny blocks and sequence overlap on the synteny score. Examples for the influence of the number of synteny blocks and different sequence overlap on the synteny score of a single hypothetical region. **a–e** Examples for the synteny scores when a single synteny block is identified, with different levels of sequence coverage. **f–i** Synteny scores of regions with 100% coverage and different numbers of synteny blocks.

Each collection of homologous ~5-kbp regions (that is, derived from a BLAST search using the same central region query) is assigned to a region-specific bin (Fig. 1c). Within each bin, we perform an all-versus-all pairwise sequence alignment to identify synteny blocks (Fig. 1d) using the DECIPHER R package^21^. Then, for each pairwise alignment, we calculate the region-specific pairwise synteny score. This score is based on two parameters: the number of synteny blocks identified in each pairwise sequence alignment and the overlap between the two sequences. The synteny score is inversely proportional to the first and directly proportional to the second (Extended Data Fig. 1).

A single synteny block in a pairwise alignment can stem from two genomic regions with a high sequence similarity. A high number of synteny blocks can result from insertions, deletions, recombination events or several SNPs located within a very close proximity in just one of the two sequences. The sequence overlap is defined as the ratio of the accumulative length of all blocks to the length of the shorter DNA region in each pairwise comparison. The region-specific pairwise synteny score has a maximal value of 1, reflecting identification of a single synteny block and overlap of 100% (Fig. 1d).

#### Step 3: calculation of the APSS

After calculating the per-region synteny scores in all bins, we randomly subsample *n* regions per single comparison of metagenomic samples (or pair of genomes) and calculate the APSS by averaging the per-region pairwise synteny scores. Pairs of samples or genomes with fewer than *n* regions per comparison are excluded from downstream analysis (Fig. 1e). By default, *n* is equal to 40, 60, 80, 100 and 200 regions per pairwise comparison.

### SynTracker is sensitive to structural variants, not to SNPs

**Fig. 2:**
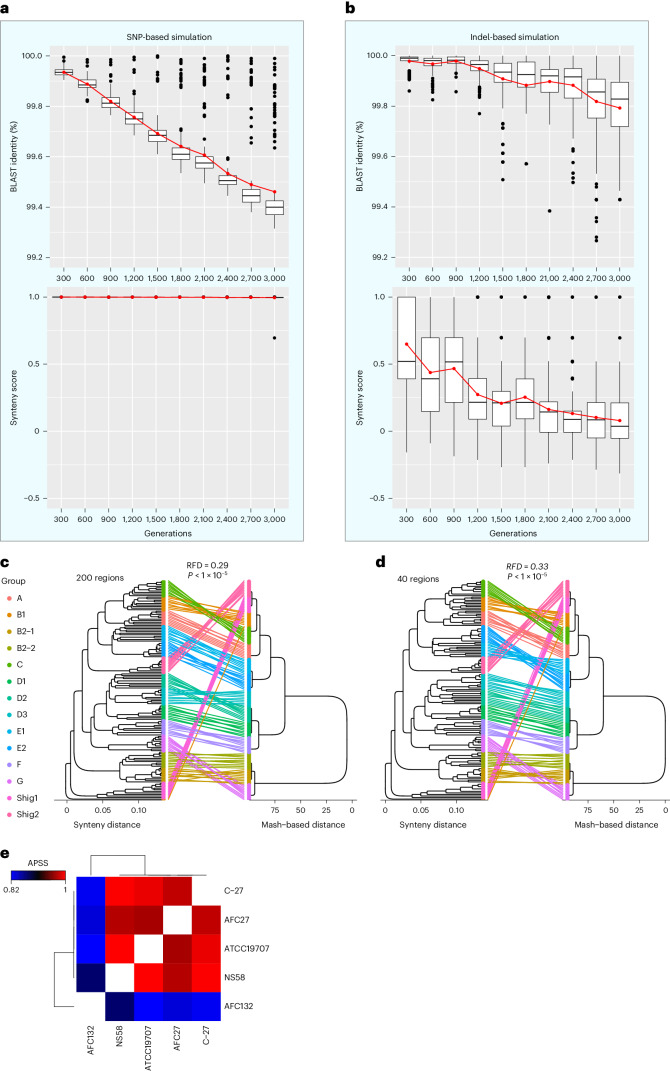
SynTracker shows robust strain-resolving performance using a small fraction of the genome length. **a**, Analysis of the genomic diversity of in silico evolved bacterial populations. Simulations were carried out for 3,000 generations through the exclusive introduction of SNPs at a frequency of 1 × 10^−6^ substitutions per nucleotide per generation. At each time point, 20 genomes were sampled and a pairwise comparison of the same 20-kbp region was performed using BLASTn (top) and SynTracker (bottom) (that is, 190 pairwise comparisons per time point). Horizontal black lines mark the group median and the red lines connect the group means (red dots). Boxes correspond to the interquartile range (IQR) and whiskers are extended to the largest and smallest observations within the first and third quartiles ± 1.5 × IQR. **b**, Same as in **a** but with simulations based on the introduction of indels at a frequency of 1 × 10^−7^ events per nucleotide per generation. **c**, Phylogenetic trees for 140 *E*. *coli* genomes belonging to 14 different phylogroups based on APSS. Left, tree based on 200 randomly selected 5-kbp regions per pairwise comparison. Right, Mash distance tree derived from the literature^24^. Colored lines connect the same genomes on both trees and lines are colored by phylogroup. *P* < 1 × 10^−5^ based on 100,000 randomizations of the synteny-based tree. **d**, Same as for **c** but using 40 regions per pairwise comparison. *P* < 1 × 10^−5^. **e**, Heat map showing the APSS of comparisons of five *N*. *oceani* strains, reflecting previously published synteny-based strain similarities^27^.

**Extended Data Fig. 2:**
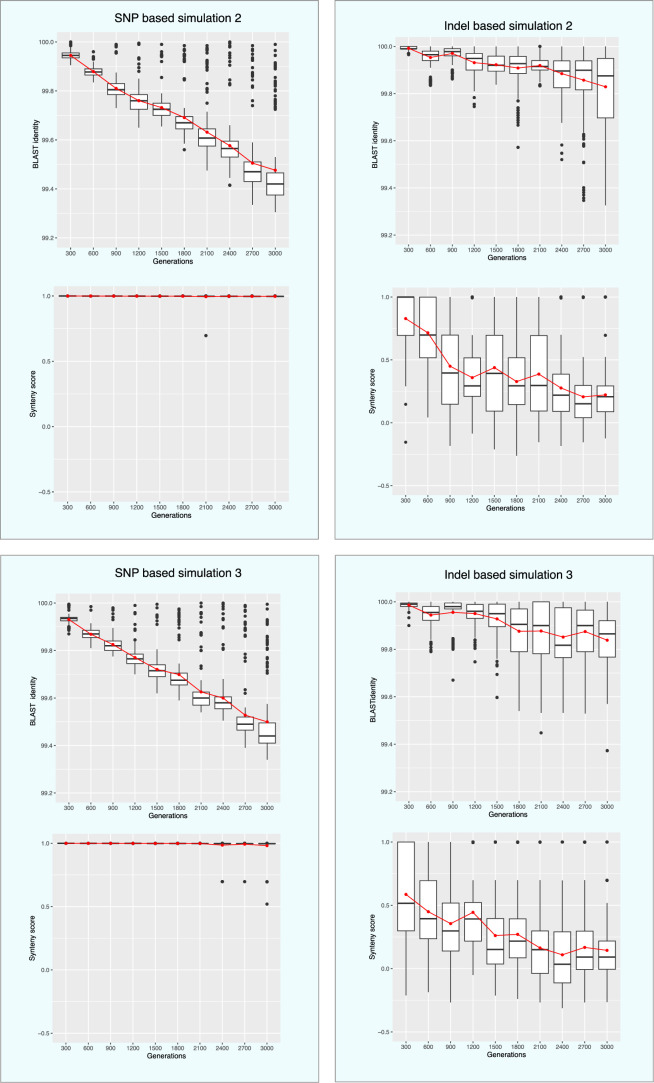
Analysis of the genomic diversity of *in-silico* evolved bacterial populations. Analysis of the genomic diversity of *in-silico* evolved bacterial populations (simulation 2 and 3, for the simulation of region 1, see Fig. 2a). Simulations were carried out for 3000 generations, through the exclusive introduction of SNPs at a frequency of 1*10^−6^ substitutions per nucleotide per generation. At each time point, 20 cells were sampled, and a pairwise comparison of the same 20 kbp region was performed using BLASTn (top panel) and SynTracker (bottom panel) (that is, 190 pairwise comparisons per timepoint). Horizontal black lines mark the group median, the red lines connect the group means (red dots). Boxes represent the interquartile range (IQR), whiskers are extended to the largest and smallest observations within the first and third quartiles +/− 1.5*IQR, correspondingly’.

We examined SynTracker’s performance and estimated the effect of different genomic variations on the synteny scores. In a first test, we used Bacmeta^22^ to generate in silico simulations of the evolution of bacterial populations. We performed two types of simulations: (1) the population was evolved by introducing SNPs exclusively and (2) only insertions and deletions were introduced. At each time point, for each genomic region, we sampled 20 ‘bacteria’ and calculated all pairwise synteny scores in addition to all pairwise sequence identities (that is, three sets of 190 pairwise comparisons at each time point; Fig. 2a and Extended Data Fig. 2). In simulations using SNPs, the minimal average BLAST identities were 99.48%, 99.46% and 99.5%, for regions 1, 2 and 3. The lowest average BLAST identities in simulations based on insertions and deletions were higher, at 99.79%, 99.79% and 99.84% (*P* < 2.2 × 10^−16^). The minimal average synteny scores in SNP-based simulations were higher (0.995, 0.995 and 0.981) than in indel-based simulations (0.103, 0.103 and 0.206; *P* < 2.2 × 10^−16^) even though the mutation frequency in SNP-based simulations was tenfold higher than the indel-based simulations. The lower synteny scores of genomic regions in the indel-based simulations highlight the higher sensitivity of the synteny-based approach to indels. These results show that populations evolving exclusively through the introduction of SNPs have a marginal reduction in the synteny scores compared to populations that evolve through the introduction of insertions and deletions at a lower mutation frequency.

### SynTracker classifies strains using a fraction of the genome

We examined the performance of SynTracker when comparing closely related genomes and assessed APSS values as the basis for the clustering of genomes into phylogenetic groups. We used a published classification of >10,000 *Escherichia* *coli* genomes based on whole-genome nucleotide content (Mash^23^), which identified 14 distinct phylogroups^24^. We randomly selected ten genomes per phylogroup and analyzed these 140 genomes eight times, randomly selecting 20–200 regions per pairwise comparison (representing ~1.8–18.5% of the *E*. *coli* O157:H7 genome length). We used the APSS values (Fig. 1e) to construct phylogenies.

**Extended Data Fig. 3:**
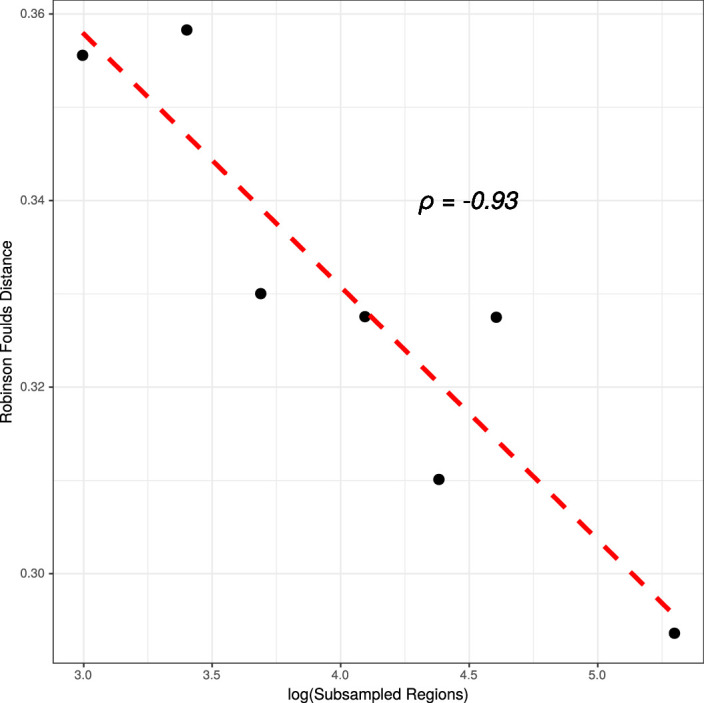
The influence of the number of regions/pair on Robinson-Foulds Distances. Correspondence between the number of regions/pairwise-comparison and the Robinson-Foulds Distance (RFD), when comparing the synteny-based and MASH-distance based phylogenetic trees. Correlation was tested using the Spearman’s rank test.

**Extended Data Fig. 4:**
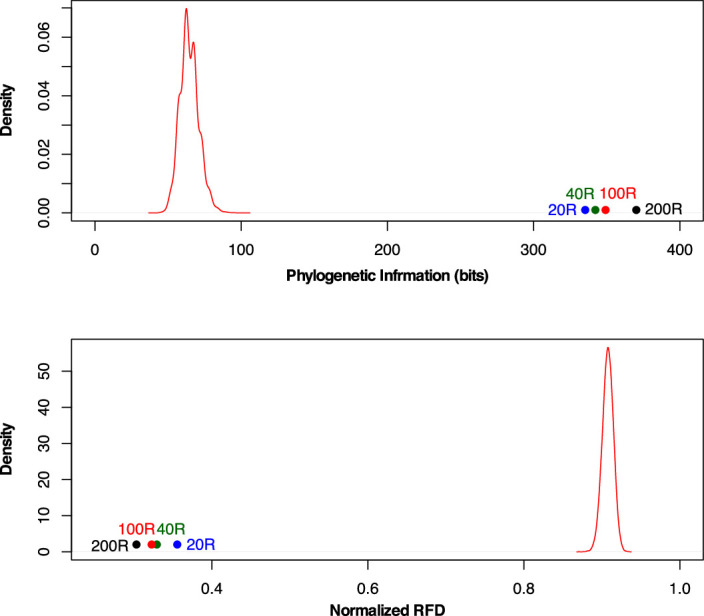
Distribution of distances between random phylogenetic trees and the MASH based tree. Upper panel shows the Phylogenetic Information content (higher values stand for higher similarity), lower panel shows the Normalized Robinson-Foulds Distance (lower values stand for higher similarity between trees). Red curves are the distribution of the distances between 1*10^5^ random trees and the MASH based tree, the points mark the distances of the actual Synteny based trees and the MASH based tree. 20R/40R/100R/200R represent the number of regions used to generate each tree.

The published Mash tree demonstrates the classification of *E*. *coli* genomes into 14 phylogroups; we checked whether we could recapitulate these phylogroups using APSS. We compared the Mash-based tree and the synteny-based trees using two methods: (1) calculating the Robinson–Foulds distance (RFD) between trees^25^ and (2) using phylogenetic information content^26^. To determine the statistical significance of these distances, we further calculated the distances (both RFDs and phylogenetic information content) between the published Mash tree and 1 × 10^5^ randomly generated trees (Fig. 2c,d and Extended Data Fig. 4). We observed that the number of sampled regions per pairwise comparison was inversely correlated to the RFDs and positively correlated to the phylogenetic information (Extended Data Figs. 3 and 4). These results indicate that, when a greater proportion of the genome was sampled by SynTracker, the Mash-based and APSS-based trees became more similar. Regardless of the proportion of the genome sampled, for both tree comparison methods, the resulting *P* value was smaller than 1 × 10^−5^ in all subsampling values. This indicates that, even with less than 2% of the genome sampled, the APSS-based tree recapitulated the published tree. Importantly, this result indicates that synteny can be used to classify *E*. *coli* strains into the ‘correct’ phylogroup using a very small fraction of the whole genome.

In a second analysis, we used data for five *Nitrosococcus* *oceani* strains isolated in different global locations^27^. The authors used a gene synteny-based analysis using genome content and alignments. Their analysis revealed that the genomes of four of the strains (C-107, NS58, C-27 and AFC27) were highly conserved in content and gene synteny, while strain AFC132 contained an additional gene repertoire and differed in gene synteny from the other four genomes. Using ~28% of the genome, our SynTracker analysis corroborated these findings; pairwise comparisons of strains C-107, NS58, C-27 and AFC27 resulted in high APSSs (0.968–0.998), while AFC132 showed a much lower APSS to the others (0.82–0.87) (Fig. 2e).

### Setting APSS thresholds for same-strain designations

In most strain-tracking software, score thresholds are used to determine whether the same strain is present in multiple samples^28,29^. To apply this concept to SynTracker results, we sought to determine an APSS threshold to use for designating two strains as the same. We note that the definition of a strain is ambiguous and highly subjective. Here, we assumed that two members of a species identified in the same individual over a time period of months likely belong to the same strain, while two members of a species colonizing different individuals likely belong to different strains. To establish an APSS threshold for the same strain for species of the human gut microbiome, we based our analysis on the longitudinal human gut metagenome^30^. We divided the dataset into training and testing sets, consisting of 117 and 106 metagenomic samples, respectively, and calculated the APSS scores for all strain pairs from 33 species in the training set. We then used the Youden statistic^31^ to determine the APSS value that optimally classified strain pairs as the same or different, which served as the basis for the thresholds used in subsequent analyses (Methods).

To check how well SynTracker performed when tracking strains, we first used it to track strains in the Poyet et al.^30^ testing set and determined the average specificity and sensitivity with each number of subsampled regions per pairwise comparison (Methods, Supplementary Table 8 and Extended Data Fig. 7). We then applied the APSS thresholds to a separate dataset to identify strain sharing between mothers and infants^32^. We observed a high proportion of strains shared between mothers and infants when infants were very young. Interestingly, as infants aged, the total number of shared strains increased, while their proportion of all pairwise comparisons decreased (Extended Data Fig. 8). These results highlight SynTracker’s utility for uncovering biologically meaningful phenomena when used as a standalone strain-comparison tool.

### Synteny-based and SNP-based analyses reveal modes of genome evolution

Given SynTracker’s high sensitivity to structural genomic variations and low sensitivity to SNPs, we aimed to use it in combination with an SNP-based strain-tracking tool to examine how each of these tools captures different types of within-species genomic diversity. We applied SynTracker in combination with inStrain, a widely used strain-tracking tool with high sensitivity to SNPs^28^. The datasets we used here were (1) a collection of 12 *Neisseria* *gonorrhoeae* clinical isolates harboring antibiotic resistance (Supplementary Table 2 and Methods); (2) a population of hypermutator *E*. *coli* isolates that emerged after the colonization of four mice with two ancestral *E*. *coli* substrains^33^ (Supplementary Table 3); (3) 77 *H*. *pylori* clinical isolates obtained from six individuals^2^ (Supplementary Table 4); and (4) a collection of *Streptomyces* *rimosus* M527 from different fermentations (Supplementary Table 5 and Methods).

**Fig. 3:**
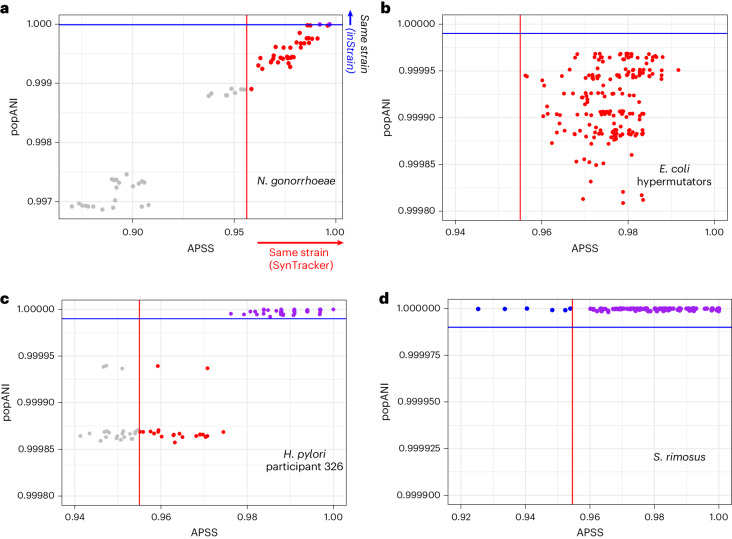
Combined synteny-based and SNP-based analysis of simple microbial populations. **a**–**d**, Points represent specific pairwise comparisons; popANI, SNP-based strain similarity, as calculated by inStrain. Red and blue lines show the same-strain cutoffs for SynTracker (>0.955) and inStrain (>0.99999); the same cutoffs are used in each panel. Purple points mark comparisons classified as the same strain by both tools, red points mark comparisons classified as the same strain by SynTrakcer, blue points mark comparisons classified as the same strain by inStrain and gray points are pairs marked as belonging to different strains by both tools. *N*. *gonorrhoeae*, *n* = 66 comparisons (**a**); hypermutator *E*. *coli*, *n* = 185 comparisons (**b**); *H*. *pylori* clinical isolates (participant 326), *n* = 91 comparisons (**c**); *S*. *rimosus* M527, *n* = 185 comparisons (**d**).

**Extended Data Fig. 5:**
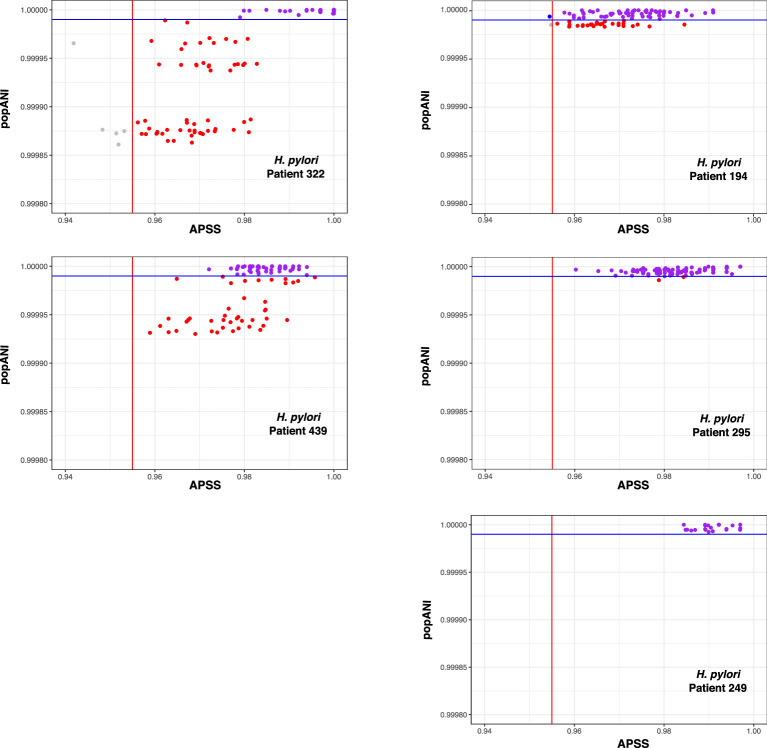
Combined SynTracker/inStrain analysis of *H.pylori* isolates. Combined SynTracker/inStrain analysis of H.pylori isolates, obtained by biopsies. Each plot shows comparisons of isolates obtained from a single patient. Points represent specific pairwise comparisons, popANI - SNP-based strain similarity, as calculated by inStrain. Red and blue lines show the SynTracker and inStrain same-strain cutoffs. Purple points mark comparisons classified as the same strain by both tools, red by SynTrakcer, blue by inStrain, gray are pairs marked as belonging to different strains by both tools. n = 91, for all patients, excluding patient 249, n = 21.

First, we performed 66 pairwise comparisons for the *N*. *gonorrhoeae* isolates. This species yielded a very high correlation between the SNP-based and synteny-based strain similarity scores (Spearman’s $\rho$  = 0.985), suggesting that the genomic diversity of this species is achieved through both point mutations and structural genomic variation (Fig. 3a). SynTracker identified a larger proportion of the comparisons as the same strain compared to inStrain. Second, for the *E*. *coli* population, we performed 185 pairwise comparisons using inStrain; none of them were classified as the same strain using inStrain’s default same-strain threshold. In contrast, the SynTracker analysis classified all as belonging to the same strain using an APSS threshold of 0.955 (slightly more stringent than the threshold used above). Third, when applied to the *H*. *pylori* genome data, we performed 21–91 pairwise comparisons per participant and observed that, in three of the participants (194, 249 and 295; Extended Data Fig. 5), both tools assigned a majority of the genome pairs to the same strains. In the three other participants (322, 326 and 439), however, we detected additional subpopulations: subsets of genome pairs classified as belonging to the same strain by only one of the tools or neither (Fig. 3 and Extended Data Fig. 5). These results indicate that, within a single species and within a single host, subsets of *H*. *pylori* are generating genomic diversity using two very different modes, sometimes together and sometimes independently. Fourth, for the *S*. *rimosus* samples, we performed 185 pairwise comparisons and observed that, while all strain pairs appeared as clonal in the SNP-based analysis, the SynTracker analysis resulted in a wide range of APSS values, with some classified as different strains. This result suggests that the within-species genomic variation of *S*. *rimosus* is achieved through structural differences but not SNPs (Fig. 3d). These combined results are consistent with inStrain’s sensitivity to SNPs and SynTracker’s sensitivity to genomic structural differences. Each method provides a different view of the within-species genomic diversity. When used in combination, they powerfully synergize to generate a more comprehensive view of the modes of evolution underway.

**Extended Data Fig. 6:**
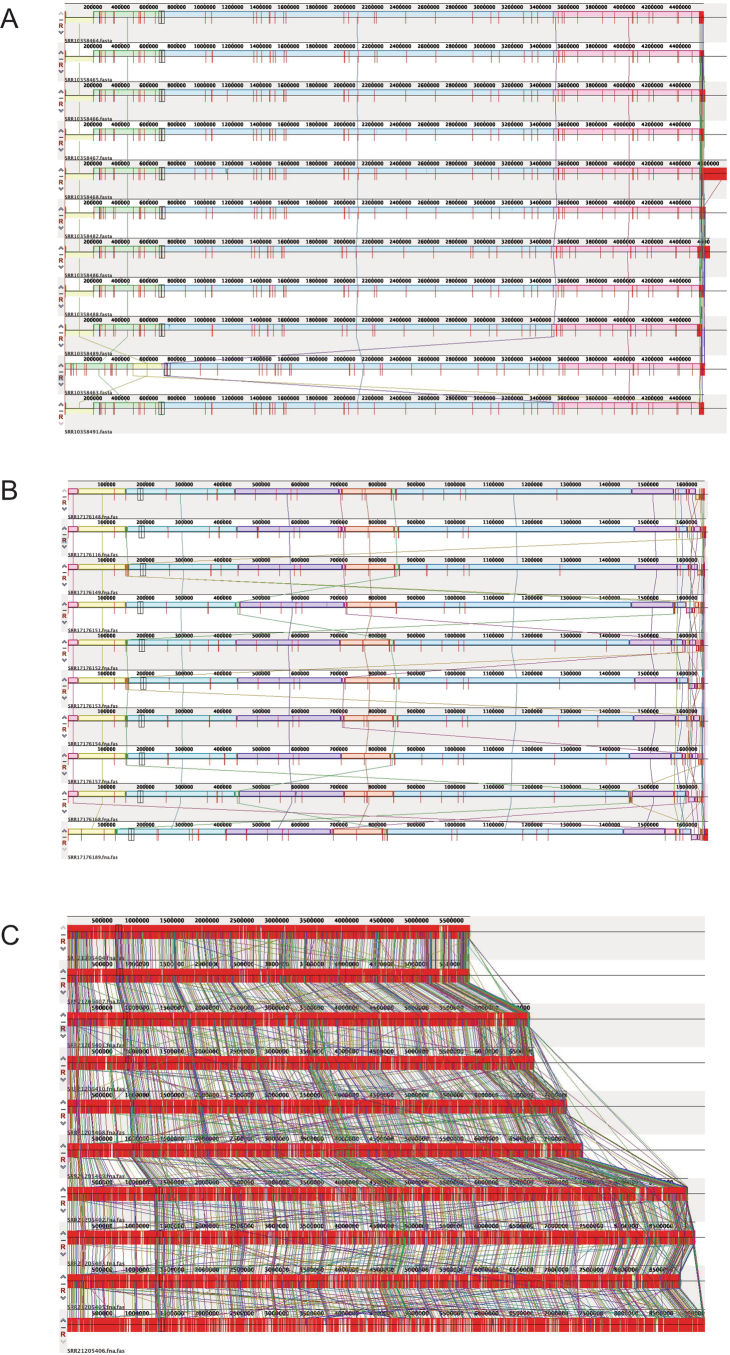
Whole genome alignments of representative assemblies analyzed by both SynTracker and inStrain. (**a**) E. coli, (**b**) H. pylori (obtained from patient 194), (C) S. rimosus.

To validate our findings, we performed genome alignments for randomly selected isolates from the *E*. *coli*, *H*. *pylori* and *S*. *rimosus* datasets. In agreement with our results, we observed that the overall genome order was more uniform in *E*. *coli* compared to *H*. *pylori* and *S*. *rimosus* (Methods and Extended Data Fig. 6).

### SynTracker applied to microbiomes reveals evolutionary patterns

We applied SynTracker to fecal metagenomes obtained from 1,133 individuals (747 adults and 386 related infants) residing in three countries^34^. In a first analysis, we used MIPs (both related and unrelated) and combined the SynTracker analysis with inStrain analysis to identify species with distinct modes of accumulating genomic diversity, that is, point mutations or insertions, deletions and recombination. In a second analysis, we excluded true MIPs and tracked strains across locations to characterize spatial patterns exhibited by different species. In both analyses, we used the same set of MAGs as a reference for both SynTracker and inStrain.

1. **Detection of hypermutators and hyper-recombinators from metagenome data.** Overall, SynTracker made twice as many strain comparisons as inStrain (40,000 versus 19,000), of which ~12,000 pairwise comparisons were performed by both tools. Despite the fact that this subset of comparisons was detected by both tools, each tool classified a different set of pairs as having the highest strain similarities (Fig. 4a). To identify which taxa comprised the sets of strains flagged as most similar by each of the tools, we compared the enrichment *P* values of each species in the two subsets (that is, most similar 5% of the strain comparisons according to each tool). Most species showed similar enrichment in both sets, suggesting that their within-species genomic diversity originated from both SNPs and structural differences. However, a subset of species showed differential enrichment in one or the other set, indicating that their genomic diversity originated preferentially from point mutations (hypermutators) or from structural differences (hyper-recombinant or increased indel rate). Hypermutators included *Phocaeicola* *vulgatus*, *B*. *fragilis* and *Alistipes* *putredinis*, while hyper-recombinators included *Phocaeicola* *massiliensis*, *Streptococcus* *thermophilus*, *Prevotella* spp., *Streptococcus gallolyticus* and others (Fig. 4b).

**Fig. 4:**
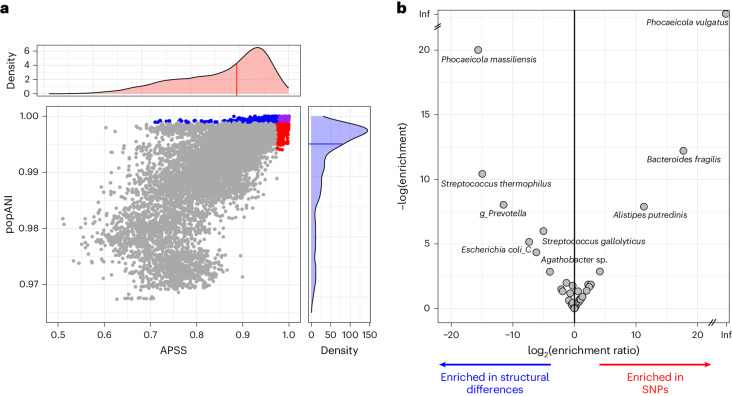
Combined synteny-based and SNP-based analysis of human gut microbiome reveals patterns of within-species genomic diversity. **a**, Synteny-based (APSS) and SNP-based (popANI) strain similarities in human gut metagenomes collected in Gabon, Germany and Vietnam. Dots represent pairwise comparisons within a species. Dots are colored when they are in the top 5% most similar: blue by inStrain, red by SynTracker and purple by both (all others are gray). Density plots show the distribution of scores for each tool. **b**, Species showing enrichment of SNPs or structural differences. Points represent species. The *x* axis shows the ratio between the enrichment of each species (that is, the hypergeometric *P* value) in the most similar 5% of the comparisons by tool. Right, species enriched in SNPs; left, species enriched in structural differences. The *y* axis indicates the degree of enrichment.
2. **Networks built from APSS values allow the visualization of strain patterns.** To visualize strain relatedness, we used APSS scores to build networks, where nodes represent hosts and edges are weighted by their APSS. In this analysis, we used SynTracker to assess whether conspecific strains sampled from persons living in geographic proximity are more similar to each other compared to strains obtained from people living further apart. In total, we were able to assess 145 species (that is, those identified with a sufficient genome coverage in >5 hosts), 61 of which showed significantly higher APSS values in individuals living in the same province (in Gabon or Vietnam) or in the same federal state (Germany), compared to individuals living in different provinces or states (Fig. 5a). For 39 species, the difference in mean APSS between strains obtained from the same or different province or state was medium to large (|*d*| > 0.5; Fig. 5a).

**Fig. 5:**
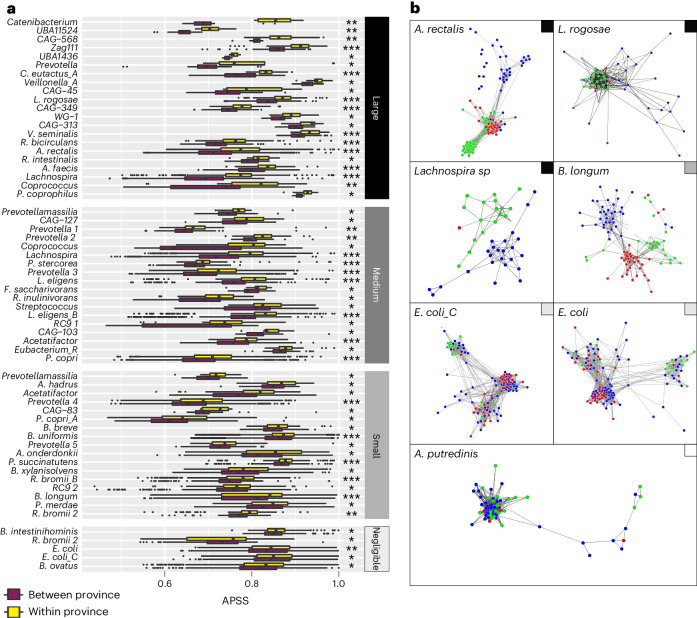
Strain analysis of human gut metagenomes collected in Gabon, Germany and Vietnam. **a**, Distribution of strain similarities for species within gut microbiomes of individuals living within the same (yellow box) or different (maroon box) provinces (Gabon and Vietnam) or federal states (Germany). Stars correspond to Benjamini–Hochberg-corrected *P* values (one-sided Wilcoxon–Mann–Whitney test). *q < 5 × 10^−2^, **q < 5 × 10^−3^ and ***q < 5 × 10^−5^. Species are sorted according to the effect size and rightmost bars denote the effect size magnitude (large, medium, small and negligible). **b**, Species-specific strain-similarity networks. Nodes represent hosts and are colored by country (red, Gabon; blue, Vietnam; green, Germany) and edges represent strain comparisons, with APSS values as edge weights. Nodes are clustered by edge weights. Colored squares point to the magnitude category of the effect size of the species, as given in **a**.

The networks highlighted strain patterns by species by country. For instance, *Lachnospira* *rogosae* and *Agathobacter* *rectalis* showed distinct strain clusters within countries (Cohen’s *d* = 1.09 and 0.99, respectively; Fig. 5b). Similarly, the strain comparisons for *Lachnospira* *sp003537285* resulted in two unconnected network clusters, one from Vietnam and one from Germany (with one sole carrier from Gabon). These results suggest the within-population evolution of strains with limited dispersal. In contrast, the genomes of two *E*. *coli* subspecies (Cohen’s *d* = 0.09) formed a network composed of distinct clusters, each containing participants from all three countries (Fig. 5b). This pattern suggests a high degree of geographic exchange across large distances. The species *A*. *putredinis* also yielded a network suggestive of cosmopolitan strains.

The network diagrams also allow subtle patterns of strain distribution to be readily identified. For instance, *Bifidobacterium* *longum* is primarily a cosmopolitan species (according to Cohen’s *d* value) and its strain comparisons resulted in three clusters, each composed of participants living in a single country. Surprisingly, a fourth cluster was made up from strains obtained from participants from all three countries. This pattern suggests the coexistence of geographically constrained and cosmopolitan strains within *B*. *longum*. Taken together, the network visualization of APSS scores provides a holistic view of strain patterns to emerge from highly complex data and the simultaneous assessment of strain dynamics. Although we applied this concept to spatial patterns here, any other type of metadata could be applied to the networks to aid interpretation of the patterns.

### Benchmarking SynTracker against other strain-comparison tools

To benchmark SynTracker against three state-of-the-art strain-comparison tools (MIDAS^35^, StrainPhlAn^36^ and inStrain^28^), we used a test performed in the literature^28^, in which these tools were used to classify strains as shared or nonshared at different average nucleotide identity (ANI) cutoffs. In this test, gut metagenomic samples obtained from three pairs of premature infant twins were used. As the ground truth, conspecific strains residing in unrelated infants were assumed to be different, while those residing in twin infant pairs were considered the same, based on previous findings^37^. In a similar manner, we used SynTracker to identify shared strains in these metagenomic samples over a range of APSS values. The reference genomes used in this analysis were the species-representative genomes (SRGs; MAGs assembled from the same metagenomic samples and clustered on the basis of ANI) obtained from the literature^28^.

**Fig. 6:**
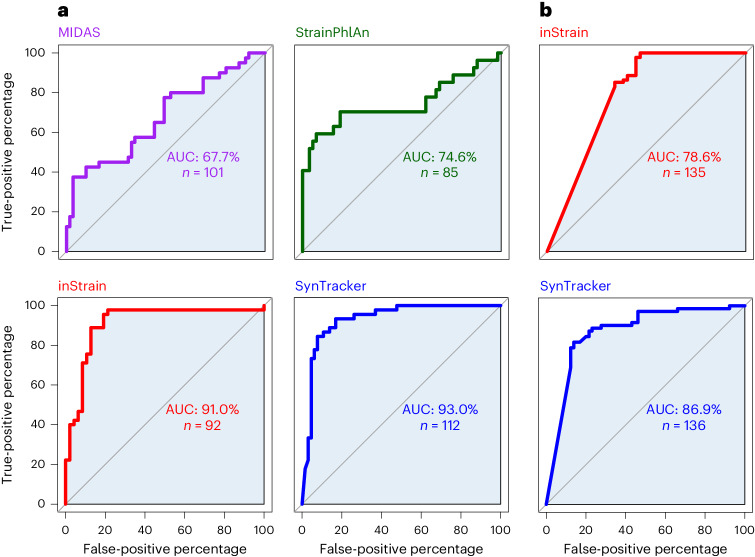
Performance of four different strain-comparison tools. **a**,**b**, Bacterial (**a**) and phage or plasmid (**b**) strain tracking when used to identify strain sharing in twin infant pairs. Test results for previously published tools (MIDAS, StrainPhlAn and inStrain) were obtained from the literature^28^. Classification as the same strain of conspecific strains residing in twin infants by each of the tools was considered as a true-positive classification, while classification of strains as the same in unrelated infants was considered as a false-positive classification. The AUC is an estimator of the method performance, in terms of the ratio of true-positive rate and false-positive rate at different ANI (MIDAS, inStrain and StrainPhlAn) or APSS (SynTracker) values. *n* represents the total number of strain-pair comparisons performed by each tool.

To determine the performance of each of the methods, we generated a receiver operating characteristic curve (ROC) for each tool and calculated the area under the curve (AUC), which is an indicator of its performance. SynTracker achieved the highest AUC value (0.93) among the previously published tools when using 40 regions per pairwise comparison (Fig. 6a). SynTracker proved to have the highest sensitivity, detecting 112 strain pairs compared to 85, 92 and 101 in StrainPhlAn, inStrain and MIDAS, respectively. Additionally, SynTracker proved to be the most effective tool when tracking plasmid and phage strains, outperforming inStrain (Fig. 6b).

## Discussion

We introduce SynTracker, a highly performant tool for comparing segments of DNA (microbial strains) using genome microsynteny. As a standalone tool, SynTracker can be used to compare genomes or genome fragments obtained from sources such as plasmids, viruses and microbiota, with applications such as microbial strain tracking. Combined with SNP-based tools, SynTracker can be used in complex microbiomes to differentiate species whose genomic diversity results from hypermutation (increased point-mutation rate) or hyper-recombination (normal point mutation rate but high levels of genomic structural differences, such as recombination, insertions and deletions). The combination of synteny-based and SNP-based comparisons is powerfully synergistic; together, these approaches can provide new insights into the mechanisms driving species and strain diversification in microbiomes.

Although genomic information has been used for decades to delineate species, there is currently no single definition for microbial species. Defining strains is equally if not more challenging. Definitions vary by context and by researcher. For example, genomes were assigned to different strains if they presented different phenotypes and differed by only a few SNPs^33^ and, in other instances, a minimal genome similarity was used to assign strains^28,35^. Because of the subjectivity inherent to classifying strains as the same or different, we recommend that researchers consider avoiding such classifications and instead assess strain relatedness using measures of genomic differences, such as the APSS presented here.

The classification of genomes into strains is sometimes necessary, however. Strain delineations used in strain tracking often use a minimal cutoff value of genomic similarity to bin strains into same or different categories^28,35,38^. Such thresholds are subjective and depend on the species being considered, as different taxa accumulate genomic differences at different rates, among other factors. For example, when analyzing longitudinal data, one challenge is to assess when a microbial lineage has accumulated enough differences compared to the ancestral population to be considered a different strain. The widely used software inStrain uses a popANI cutoff of 99.999%, which is based on comparisons of strains in (1) mock communities and (2) the same infant over a few days to a few weeks^28^. Similarly, we set an APSS score cutoff of 0.95 to designate the same strain in the human gut on the basis of an analysis of the APSS of genome pairs obtained from longitudinal samples from the same or different hosts. This was based on the assumption that the same strain persists in the gut of healthy adult individuals over a time period of weeks to months, while conspecific strains inhabiting unrelated individuals likely belong to different strains.

It is important to note that this APSS cutoff was based on averaging different per-species cutoffs and may not be optimal for all settings. The researcher needs to consider their context and question and set the appropriate cutoff. Factors to consider include the evolvability of the microbial species, the type and magnitude of selection and the user’s interpretation of the term ‘strain’. We encourage users analyzing nontypical microorganisms or those who wish to define APSS cutoffs tailored to their system to perform an analysis similar to our analysis of the dataset in the literature^30^ using a training dataset relevant to their research question.

We previously applied SynTracker to gut metagenomes from MIPs to show strain sharing between MIPs and community strain transmission^34^. Applied to the mother–child metagenome dataset in the literature^32^, results from SynTracker largely corroborated those obtained from SNP-based tools for the strain persistence of early-colonizing species in the infant gut^39,40^ and the acquisition of strains during late infancy^35,41^. Our analysis of metagenomes from geographically distinct hosts^34^ combined with network-graph analysis based on synteny distances revealed that a notable portion of the species we assessed are made up of strains with a limited geographic distribution, indicating that they are local to a population. Intriguingly, some species contained both geographically distinct and cosmopolitan strains.

Another powerful way to use the output of SynTracker is in combination with that of an SNP-based tool, such as inStrain. Combined, these approaches readily reveal differences between species in the nature of their genomic diversity. The genome of the human pathogen *N*. *gonorrhoeae* displays both point mutations and structural changes, such that the similarities of strain pairs according to the two methods is highly correlated. This is consistent with the ease of recombination and variable genome reported for this species^42^. Similarly, *H*. *pylori*, a species with potentially both a high rate of genome rearrangement and a high mutation frequency^43,44^ displayed both in our combined analysis. This was also the dominant pattern we observed for the majority of human gut microbiome species.

The combined use of SynTracker and inStrain allowed us to detect species with outlier behavior: those enriched in either structural differences or mutations. A single-species example is *S*. *rimosus*, in which we observed a high level of structural differences with a low level of point mutations. In accord, *Streptomyces* are known for high rates of recombination^45–47^. Our analysis of the human microbiome revealed a few species with this hyper-recombinator phenotype, from several phyla: Bacteroidota, Bacillota and Pseudomonadota. Two belonged to the genus *Streptococcus*, which was also previously found to evolve through greater recombination relative to mutation^48,49^. In contrast, our combined analysis of single species revealed *E*. *coli* as a hypermutator, in line with previous observations^33,50^. In the human gut microbiome, hypermutator species included *A*. *putredinis*, *B*. *fragilis* and *P*. *vulgatus*. All are members of Bacteroidales. Of these, only *B*. *fragilis* has been studied for its mode of evolution and the results were consistent with our observations^1^. Generally, our single-species observations are consistent with published studies. The synergetic combination of synteny-based and SNP-based tools allows the simultaneous observation of potentially thousands of species from a metagenome.

The combined use of SNP-based and synteny-based analyses will allow researchers to delve into the phylogenetic and environmental contexts underlying higher-than-expected mutation or recombination in select taxa. *E*. *coli* grown in the laboratory displays high rates of point mutation^33,50^. In our analysis of human gut metagenomes, however, *E*. *coli* was revealed to preferentially undergo recombination. Although these differences in how genomic diversity is generated may be attributed to the different subtypes of *E*. *coli*, these results illustrate the potential of combining synteny-based and SNP-based approaches to unravel the forces operating on species to drive one mode of evolution over another. Using synteny to discern strains undergoing high levels of recombination may have practical implications as functional differences can emerge from recombination and/or mutation. For instance, the respiratory pathogen *S*. *pneumoniae* develops antibiotic resistance through recombination^6^. Phase variation in many important bacterial pathogens can also occur through reversible recombination events, generating subpopulations that escape immune recognition^8–10^. Thus, high recombination with low levels of mutation may also lead to important functional differences between strains, motivating the use of SynTracker to identify subpopulations that would be missed using ANI-based tools alone.

SynTracker can be used reliably as a standalone tool, similarly to other strain-tracking methods, while offering a number of advantages. We demonstrated SynTracker’s ability to track strains using only a fraction of the complete genome (that is, 2% of *E*. *coli* genome), while most other tools require coverage of 50% or more of the genome length. Moreover, benchmarking of SynTracker against state-of-the-art strain-comparison tools showed that SynTracker outperformed MIDAS^35^ and StrainPhlAn^36^ and achieved a slightly better performance score than InStrain^28^ in terms of accuracy, while outperforming all tools in terms of sensitivity (that is, the number of strain comparisons identified). Likely because of the fact that it can work well with limited data, SynTracker achieved a significantly higher AUC than inStrain when applied to phages and plasmids. Thus, when a single tool is to be used, SynTracker is particularly recommended for phages, plasmids and other low-data-input applications, such as tracking strains of rare taxa.

## Methods

### SynTracker pipeline

The SynTracker pipeline consists of three main parts. In the first part, SynTracker accepts a collection of reference genomes (a single genome per species), either fully assembled or as a collection of contigs. Each per-species reference is fragmented into a collection of 1-kbp central regions, which are binned and stored together.

In the second part, SynTracker creates a BLAST database based on a user-provided collection of metagenomic assemblies or genomes. Next, it performs a BLAST search, for each of the central regions, against the newly created BLAST database with a minimal identity of 97% and a minimal query coverage of 70% (that is, 700 bp). In the final step of this part, hits for each BLAST search are retrieved, in addition to a 2-kbp region on each side of the BLAST hit using the ‘blastcmddb’ command. Hits within <2 kbp both upstream and downstream to the hit are excluded from further analysis. Each retrieved sequence is denoted by its sample of origin and matching region in the reference genome.

In the third part of the pipeline, genomic fragments are grouped by their matching region in the reference genome and pairwise alignments are conducted to identify synteny blocks in each pair of sequences. The identification of synteny blocks in each pairwise alignment is performed using the ‘FindSynteny’ function in the DECIPHER R package^21^, with parameters ‘maxGap’ and ‘maxSep’ both set to 15. Additionally, only pairwise comparisons with a minimal overlap of 4,800 bp are considered for downstream analysis. Next, per each pairwise alignment, a synteny score is calculated:

$${\rm{synScore}}={1}+{{{\log }}}_{{10}}\left(\left({\rm{Ov}/{len}}\right)/{B}\right)$$

where Ov stands for the accumulative length of the overlapping synteny blocks identified in the pairwise alignment, len denotes the length of the shorter sequence in each pair and *B* stands for the number of synteny blocks identified in each pairwise alignment.

In the final step of the third part of the pipeline, for each reference genome, *n* genomic regions are randomly selected per pair of metagenomic samples or genomes. The APSS is calculated by averaging the individual pairwise synteny scores. Pairs of samples or genomes with fewer than *n* regions are excluded from downstream analysis.

### In silico evolutionary simulations

Calculation of the synteny scores per group of sampled cells was performed as described above. However, as the length of the genomic fragments used in the simulation was limited to 20 kbp, synteny scores were based on a single alignment of the ~20-kbp region per pair of simulated genomes. Statistical significance of the differences between BLAST identities or synteny scores in the indel-based and SNP-based simulations was performed by applying the Welch *t*-test on the entire set of pairwise comparisons at the specified above time point.

### Classification of bacterial genomes

Calculation of APSS values for *E*. *coli* strain pairs was performed as described above and using the *E*. *coli* str. K-12 substrain MG1655 genome as a reference (National Center for Biotechnology Information (NCBI) reference sequence: NC_000913.3).

Synteny-based phylogenetic trees were constructed using *n* regions per pairwise comparison (*n* = 20–200). For each number of regions per pairwise comparison, APSS values were converted to synteny distances, which are equal to the absolute value of APSS − 1. All pairwise synteny distances were placed in a symmetric matrix that was used to calculate unweighted pair group method with arithmetic mean phylogenetic trees using the ‘phangorn’ R package^51^.

The full Mash distance-based phylogenetic tree, including >10,000 genomes, was imported from the literature^24^ (‘Supplementary_Data_Fig_3c_10k_newick.nwk’ file). Tree labels were converted from MD5 to assembly accession, followed by pruning of the Mash-based tree so that only external nodes corresponding to the 140 *E*. *coli* genomes analyzed with SynTracker were kept.

The distance between each of the synteny-based phylogenetic trees and the Mash-based tree was determined by calculating the Information-based generalized RFDs using the ‘TreeDist’ R package^52^.

The significance level for each of the tree comparisons was calculated by shuffling the labels of the synteny-based tree (100,000 iterations for each comparison) and calculation of the fraction of the randomized trees with an RFD equal to or lower than the distance between the actual synteny-based tree and the Mash-based tree.

### Strain comparisons in experimentally evolved *E*. *coli* populations

*E*. *coli* sequencing libraries^33^ were downloaded from the NCBI Sequence Read Archive (SRA) (Supplementary Table 4) and were quality-filtered as described previously^53^. Sequencing libraries were assembled using the SPADES genome assembler^54^. Nonhypermutator isolates (bearing only few mutations compared to the reference genome) were excluded from downstream analysis. For both inStrain and SynTracker analyses, the same reference genome was used, as described in the literature^33^. In the SynTracker analysis, 200 regions per pairwise comparison were used.

### Strain comparisons of *H*. *pylori* clinical isolates, *N*. *gonorrhoeae* isolates and *S*. *rimosus* isolates

A total of 77 sequencing libraries of clinical *H*. *pylori* isolates^2^, 20 libraries of *S*. *rimosus* isolates and 12 libraries derived from clinical isolates of *N*. *gonorrhoeae* were obtained from the NCBI SRA database (Supplementary Tables 3 and 5), quality-filtered and assembled as described above. For both inStrain and SynTracker analyses, the same reference genomes were used: *H*. *pylori*, GenBank: CP032479.1; *S*. *rimosus*, GCF_000331185.2_ASM33118v2; *N*. *gonorrhoeae*, GCF_900087635.2. In the SynTracker analysis, 200 regions per pairwise comparison were used, except for *N*. *gonorrhoeae*, where 60 regions per pairwise comparison were used.

### Whole-genome alignments of *H*. *pylori, N*. *gonorrhoeae* and *S*. *rimosus* assemblies

Whole-genome alignments were performed using the Mauve software^55^. First, the contigs of each assembly were ordered using the reference genomes described above and then aligned against each other using the software’s default parameters.

### Combined SNP-based and synteny-based analysis of human gut metagenomes

For the combined analysis, we further used the recently published inStrain and SynTracker analyses of infant–adult pairs^34^. To create the APSS versus popANI plot, we used the subset of strain comparisons identified by both tools (>12,000 strain pairs). The SynTracker analysis was performed using 30 regions per pairwise comparison.

To identify species with a preferential mode of within-species genomic diversity, we first calculated the enrichment of each species at the 5% most similar strain pairs according to inStrain (popANI used as the distance matrix) and SynTracker (APSS). The enrichment of each species was determined using the hypergeometric distribution probability test:

$${p}({X}={k})=(({K}/k)(({N}-{K})/(n-k)))/({N}/n)$$

where *N* is the number of pairwise comparisons in each dataset, *n* is the number of the most similar pairwise comparisons (that is, 5% of *N*), *K* is the number of pairwise comparisons in each species in the entire dataset and *k* is the number of pairwise comparisons in each species in the most similar subset.

### Determining SynTracker’s performance

For each of the 33 studied species, we used a publicly available reference genome (Supplementary Table 9), which was fragmented into a collection of 1-kbp central regions, as described above. Next, we performed a per-sample, de novo metagenomic assembly to construct our search space (Methods and Fig. 1). The metagenomic assemblies were divided randomly into training and testing sets (117 and 106 samples, obtained from 45 and 43 donors, respectively). For both sets, we calculated eight different final APSS matrices per species after randomly selecting *n* regions per pairwise comparison (*n* = 15–200 5-kbp regions). We then classified pairwise comparisons in the training set to those originating from the same host at different time points (within-host set) and those originating from different hosts (between-host set). With the classification of pairwise comparisons in the training set used as ground truth, we generated ROCs^56^ to determine the specificity and sensitivity of the classification at different APSS values. We based our curves on 100 iterations for each combination of species and subsampling value.

**Extended Data Fig. 7:**
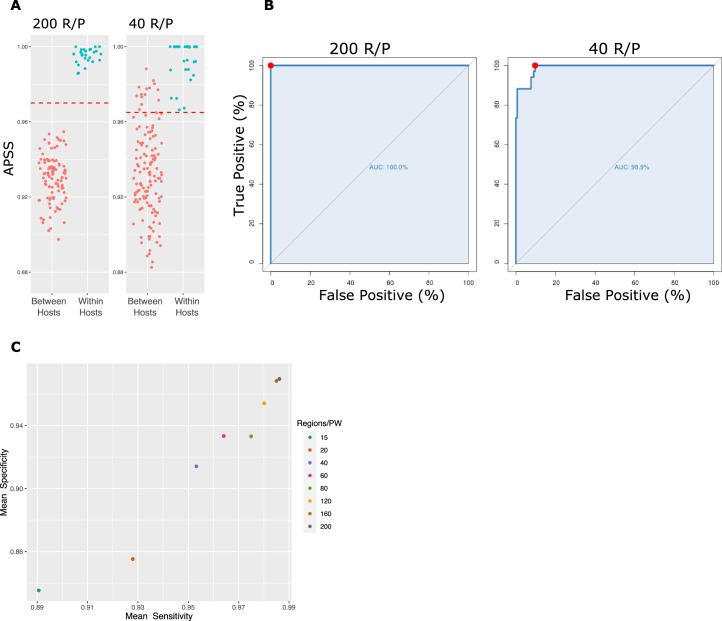
Using longitudinal human metagenomes to determine Syntracker’s performance. **a**. Representative synteny-based analyses of *B. fragilis* strains in longitudinal metagenomic samples, obtained from the same host (cyan) and different hosts (pink), using 200 and 40 regions/pairwise-comparison. Dashed red lines represent the APSS threshold differentiating between strains residing in the same host and strains residing in different hosts, as calculated in B. **b**. ROC plots of the same analyses shown in A. Red dots correspond to the Youden point and give the APSS score that yields the optimal combination of specificity and sensitivity. **c**. The mean specificity and sensitivity of the synteny approach, calculated at different numbers of regions/pairwise-comparison. R/P- regions/pairwise comparison, PW - pairwise.

**Extended Data Fig. 8:**
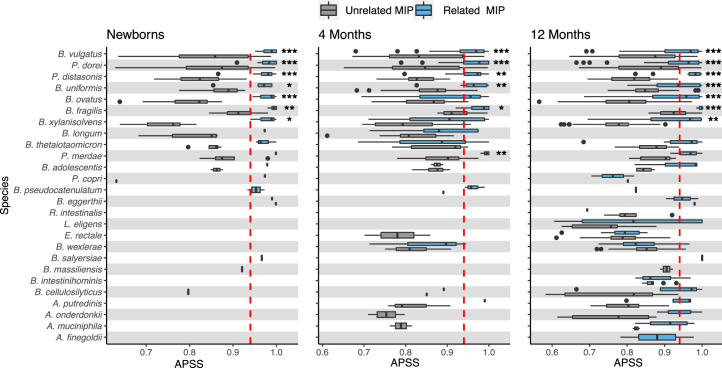
Strain sharing in Mother-Infant Pairs. The Synteny of strains residing in mothers and their infants at ages 4 days (‘newborns’), 4 months, and 1 year (Related MIP, blue boxes) was compared with the synteny of strains in unrelated mothers and infants (unrelated MIP, gray boxes). Comparisons to the right of the dashed red lines are considered strain sharing events. Stars correspond to Benjamini-Hochberg corrected p-values (one sided Wilcoxon-Mann-Whitney test): q-value * <5 × 10-2, ** < 5 × 10-3, *** <5 × 10-5. Vertical lines within boxes represent the median of each group, Boxes represent the interquartile range (IQR), whiskers are extended to the largest and smallest observations within the first and third quartiles +/− 1.5*IQR, correspondingly.

We next aimed to assess the performance of SynTracker using the 33-species test set. We first determined the APSS values that optimally discriminated within-host comparisons from between-host comparisons. Pairwise comparisons with APSSs higher than these ‘optimal’ values were considered as same-strain comparisons, while those below them were considered as distinct strains. This was achieved by calculating the *J* index^31^ for each combination of species and subsampling depth. We defined the ‘threshold APSS’ for each sampling level as the average of optimal APSS for all species for a given number of genomic regions per pairwise comparison used. Finally, we used these APSS thresholds to determine the specificity and sensitivity of our method by introducing them to the testing set. To avoid sampling bias, we repeated this process 100 times for each combination of species and sampling level and calculated the average specificity and sensitivity values. As expected, we observed a direct correspondence between the number of subsampled regions and sensitivity and specificity (Extended Data Fig. 7 and Supplementary Table 8), with maximal average sensitivity and specificity values of 98.6% and 97% for comparisons calculated using 200 regions per pairwise comparison. While a small number of regions per pairwise comparison mostly result in lower accuracy, the decision to use such values may be justified by the inclusion of additional samples in the analysis. The researcher can decide whether to prioritize increased accuracy or sample size.

Longitudinal metagenomes were obtained from the NCBI SRA database and were quality-filtered as described previously^53^. Metagenomic samples were de novo assembled using metaSPAdes^54^, with a maximal number of 20 million reads per sample. ROC curves and matching APSS thresholds for each combination of species and sampling depth in the training set were calculated using the R programing language ‘pROC*’* package^57^. Best threshold values were calculated by aggregating the results of 100 iterations for each combination of species and sampling depth. The reported specificity and sensitivity were calculated on the basis of 100 iterations per combination.

### Benchmarking SynTracker

ROC plots for MIDAS, StrainPhlAn and instrain were based on the analyses of microbiomes from six premature infants conducted in the literature^28^. The original table was filtered to exclude phage and plasmid comparisons performed using inStrain to reflect the results shown in the previous study^28^.

To benchmark SynTracker, we first downloaded the same raw-read files used for the tests performed by the authors^28^. Quality filtration and de novo assembly were performed as described above. To allow a more accurate comparison to other tools, we used the SRGs (that is, genomes representing a cluster of MAGs with ANI > 99%) assembled by the authors^28^ as reference genomes input to SynTracker. ROC plots were prepared as described above.

### Mother–infant strain transmission

Metagenomic samples were downloaded from the NCBI SRA database and were quality-filtered and assembled as described above. However, as only one of the two matching read files passed our quality filtration for some samples, we performed the metagenomic assembly using single-end reads.

### Strain similarities in different geographic locations

To identify location-specific strains, we used metagenomic samples collected in Gabon, Vietnam and Germany. Sample collection, metagenomic assembly and clustering of MAGs into SRGs were performed as described previously^34^. For our search space (Fig. 1), we used the collection of paired sample assemblies; as the reference genomes, we used the previously generated SRGs. APSS was calculated using 80 5-kbp long regions per each pairwise comparison, yielding 156 species with sufficient coverage.

To avoid the influence of mother–infant strain transmission on the within-region synteny scores, we filtered out strain comparisons between mothers and their own infants. We also removed subjects who were not born in the country in which they currently resided to eliminate comparisons between the local population and expats. The location of the subject was based on the residence place as supplied by each subject.

The statistical significance of the differences in strain comparisons within and between provinces was determined by the Wilcoxon–Mann–Whitney test, with test results corrected for multiple testing using the Benjamini–Hochberg correction, as described above. Matching effect sizes (Cohen’s *d*) were calculated and classified using the ‘effsize’ R package (v.0.8.1)^58^. Species-specific networks were generated using the CLANS software^59^, with −log_10_(APSS) used as edge weights. Minimal APSS scores to be considered in the analysis were ~0.75. Singletons (that is, subjects with no connections in the network) were removed from the network graphs.

### Recommended use of reference genomes

As state-of-the-art methods for the assembly of genomes from metagenomes yield ever-larger collections of MAGs, we propose to follow the inStrain program workflow^28^, in which the MAG collection is clustered to create SRGs to be used as the reference genomes in our pipeline. This approach should allow expansion of the study strains for novel species.

### Reporting summary

Further information on research design is available in the Nature Portfolio Reporting Summary linked to this article.

## Online content

Any methods, additional references, Nature Portfolio reporting summaries, source data, extended data, supplementary information, acknowledgements, peer review information; details of author contributions and competing interests; and statements of data and code availability are available at 10.1038/s41587-024-02276-2.

## Supplementary information

Supplementary Information Supplementary discussion.

Reporting Summary

Supplementary Tables 1–9

## Data Availability

Datasets used in this paper are specified in the Supplementary Information.

## Code availability

The SynTracker pipeline is available at https://github.com/leylabmpi/SynTracker.

Custom code used for downstream analysis is available at https://github.com/leylabmpi/Synteny_analyses.
