# Supplementary material for: Strain tracking in complex microbiomes using synteny analysis reveals per-species modes of evolution
Source: Nat Biotechnol. 2024 Jun 19;43(5):773–83. doi: 10.1038/s41587-024-02276-2 (PMC12084154; doi:10.1038/s41587-024-02276-2)
Supplement: Supplementary file 1 — Supplementary discussion. [file 41587_2024_2276_MOESM1_ESM.pdf]

nature biotechnology

Supplementary information https://doi.org/10.1038/s41587-024-02276-2

Strain tracking in complex microbiomes using synteny analysis reveals per-species modes of evolution

In the format provided by the authors and unedited

Supplementary text

The influence of region length on Average Pairwise Synteny Score (APSS):

The SynTrakcer pipeline is based on pairwise alignments of homologous sequences in pairs of genomes or metagenomic sequences. By default, the length of the regions aligned is 5 kbp, consisting of the “central region” (see Fig. 1), which is 1 kbp long, and the “flanking regions” consisting of two 2 kbp long regions located upstream and downstream to the central region. The length of the regions can be modified as desired using command line options.

Change in the length of the flanking region will result in a different distribution of APSS: this happens because the per-region synteny score is based on the number of synteny breaks identified per-region. When longer regions are analyzed, the probability of identifying more breaks per-region increases (see Equation 1).

To demonstrate how the length of the regions influences the distribution of APSS, we analyzed the data used for the benchmarking of the tool (Figure 6) and implemented 4 different region lengths: 2500 bp, 4000 bp, 5000 bp and 7500 bp. Next we plotted the region specific synteny scores, to demonstrate how the score changes as a function of length (Fig. ST1).

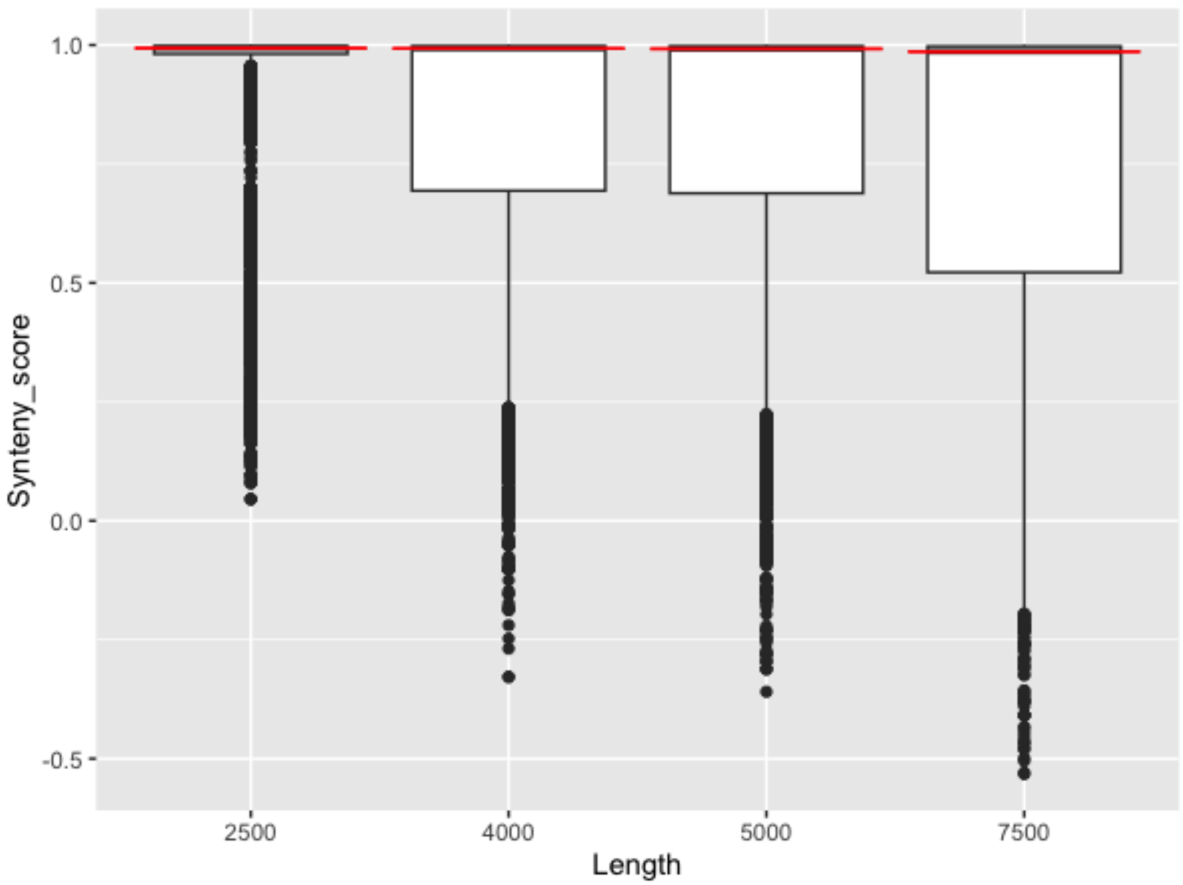

Figure ST1: Distribution of single region Synteny scores, as a function of the region length. *Data analyzed are from metagenomic samples obtained from premature newborn twins (Fig. 6). Regions with a Synteny score of 1 were removed from the analysis. Red lines stand for the group medians.*

Moreover, a longer region length will probably yield a lower number of regions compared per pair of genomes or metagenomic samples. This happens because:
(a) the entire reference genome is divided into a smaller number of regions, and (b) the probability of identifying homologous regions of suitable length decreases.

In general, we recommend using the default region length, and if possible, maximizinging the number of regions subsampled per pairwise comparison. However, if poor assemblies are being compared, it is possible to use shorter lengths to increase the number of regions compared per pair of samples.

To verify that SynTracker performs well when using different region lengths, we analyzed the data used for the benchmarking of the tool (Fig. 6), implementing 4 different region lengths: 2500 bp, 4000 bp, 5000 bp and 7500 bp. While the number of comparable strain pairs was indeed inversely correlated to the region length, the performance of SynTracker remained high in all tests (Fig. ST2).

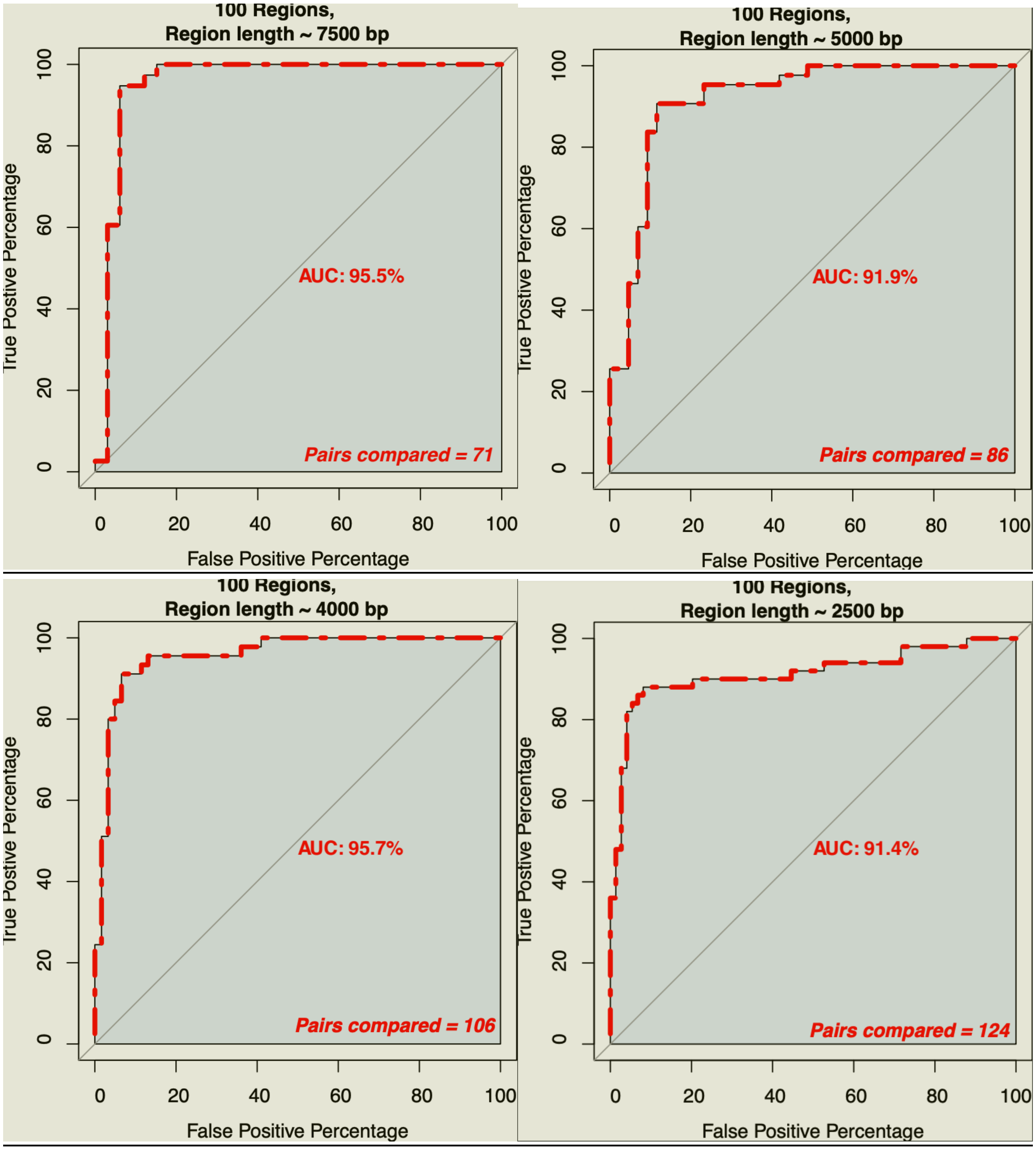

<u>Figure ST2:</u> SynTracker analyses are robust to different region lengths. *Data analyzed are the premature twin data used to benchmark SynTracker against other tools (Fig. 6). All ROC plots were created using 100 regions/pairwise comparison, at region lengths of 7500bp, 5000bp, 4000bp and 2500bp. The number of pairs compared stands for the number of strain pairs with at least 100 aligned regions.*

<u>Combined influence of the number of synteny blocks and sequence overlap on the per-region Synteny scores.</u>

The synteny score we defined in this study is influenced by two main factors: the number of synteny blocks in each alignment and the amount of overlap between the two aligned

sequences (*i.e.*, the cumulative length of the synteny blocks divided by the length of the shorter sequence of the two aligned sequences). Due to the way in which the per-region synteny score is calculated (Equation 1), the influence of the number of the synteny blocks on the synteny score is higher, compared to the sequence overlap (Fig. S1). In some cases the pairwise alignment could identify only a single synteny block, encompassing <50% of the sequence length, making the synteny score equal (or smaller) than the score of an alignment with ~100% sequence overlap and two synteny blocks (*i.e.*, a small indel is located in one of the sequences).

While this could be perceived as confusing by some users, it is important to emphasize two points: (1) When only a single block is identified, it is reasonable to assume that the non-aligned sequence extends into the adjacent region, practically lowering the synteny scores of two consecutive regions, potentially reducing the APSS; and (2) these events are very rare and have a negligible effect on the analysis results. To validate this point, we checked for the percentage of per-region alignments resulting in a single synteny block and <50% overlap between the sequences in three datasets analyzed in this work: *S. rimosus* M527, *E.coli,* and *N. gonorrhoeae* (Fig. 3). We observed that the frequencies of such events in these datasets are 0.018 %, 0.012% and 0%, respectively. Thus, only 1 out of any 10000 per-region pairwise comparisons will be affected by this potential issue. To further illustrate how little effect this has on the outcomes of the SynTracker analysis, we can assume that 100 regions are used for each APSS calculation: under this condition, such regions with a single block and low coverage, represent at most a deviation of 0.003 in the APSS for one out of 100 sample pairs.

<u>Influence of number of regions used for APSS calculation on the analysis outcomes:</u>

To calculate the APSS we subsample a number of single-region pairwise comparisons and average, per each pair of samples, the synteny scores. Genome pairs with less than the number of regions sampled are excluded from downstream analysis. As expected, the performance of SynTracker improves when using a higher number of regions, both in terms of sensitivity and specificity (Figs 2, S3, S4, S7). However, when analyzing metagenomic samples, as the number of regions/pairwise comparison increases, fewer strain pairs could be analyzed.

It could be argued that in highly diverged strain pairs, only the conserved core genome will be aligned, resulting in a low number of per-region comparisons. On the other hand, less diverged strain pairs will have a greater number of alignable regions, but those belonging to the non-core genome will have lower synteny scores. This would result in lower APSS values for less diverged strains, while more diverged strains would have high APSS values.

To understand why this scenario does not reflect the actual behavior of our tool, we can use the analysis of *E. coli* genomes, presented in Figure 2. *E.coli* has been classified into 14 phylogroups and has a famously large accessory (pan)genome. In our analysis we analyzed both diverged strains (between phylogroup comparisons) and less diverged strains (comparing within a phylogroup). We used 140 genomes (10 per phylogroup) in the analysis and we were able to generate the expected tree with 200 regions per comparison, equal to roughly one

quarter of the full genome length. We also recovered the expected tree when we dropped the number of regions down to 40 regions (and other #regions, see Fig. S4). When using a higher number of regions, we did not observe exclusion of genome pairs due to lack of alignable regions between diverged strains.

The inverse correlation between the number of subsampled regions and the number of detected strain-pairs is present mostly in analysis of metagenomic data. This is likely the result of poor assemblies, most likely stemming from low abundance of some species in a sample.

We suggest that users decide how many regions per pairwise comparison should be used based on the structure of their data and the research question. As a rule of thumb, it is recommended to use the highest number of regions per pairwise comparison, as long as no there is no significant reduction in the total number of pairwise comparisons. In analyses prioritizing a higher number of pairwise comparisons (for example comparing distributions of scores in different populations) over the accuracy of specific pairwise comparisons, we suggest using a lower number of regions per pairwise comparison, to increase the statistical power of the analysis.
